# Limitations of Daily Step Count for Assessing Health in Older Adults: The Need to Consider Walking Intensity

**DOI:** 10.3390/epidemiologia7010024

**Published:** 2026-02-05

**Authors:** Pedro Ángel Latorre-Román, Ana de la Casa-Pérez, Juan Antonio Párraga-Montilla, Jesús Salas-Sánchez, Manuel Lucena-Zurita, José Carlos Cabrera-Linares

**Affiliations:** 1 Departamento de la Expresión Musical, Plástica y Corporal, Universidad de Jaén, 23071 Jaén, Spain; platorre@ujaen.es (P.Á.L.-R.); anadelacasaperez@gmail.com (A.d.l.C.-P.); jccabrer@ujaen.es (J.C.C.-L.); 2 Facultad de Educación, Universidad Autónoma de Chile, Temuco 1090, Chile; jesus.salassanchez@unir.net; 3 Facultad de Humanidades y Ciencias de la Educación, Universidad Internacional de la Rioja, 26006 Logroño, Spain; manuel.lucenazurita@unir.net; 4 Escuelas Profesionales de la Sagrada Familia, 23400 Úbeda, Spain

**Keywords:** physical activity, step count, physical function, older adults

## Abstract

Background/Objectives: This study explored the association between daily step count (DSC) and health outcomes in older adults in Spain. A total of 668 individuals aged 60–100 years (mean = 71.33 ± 8.11 years) participated. Methods: Participants wore a Xiaomi Mi Band 4 accelerometer continuously for seven days. Physical and cognitive tests were conducted, along with questionnaires on depression, quality of life, and physical activity. Results: On average, men walked 8919.08 ± 4455.65 steps/day, significantly more than women (7855.46 ± 7855.46 steps/day, *p* = 0.002). A moderate negative correlation was found between age and DSC (r = −0.460, *p* < 0.001). The coefficient of variation in DSC increased across age groups, indicating growing heterogeneity with advancing age. Individuals in the high International Physical Activity Questionnaire (IPAQ) category walked 1517 more steps/day than those in the low activity group (*p* < 0.001), confirming IPAQ level as a strong determinant of physical activity. Participation in organized physical activity was associated with an additional 909 steps/day (*p* = 0.004). Meeting age-specific step recommendations is associated with better anthropometric, psychosocial, and cardiometabolic markers, but many of these differences disappear after adjusting for age and sex. Conclusions: DSC in older adults is strongly influenced by age, sex, and physical activity level. DSC may not adequately assess health in older adults. Walking intensity should be considered for accurate evaluation.

## 1. Introduction

According to EUROSTAT in 2020 [1], Spain presents one of the highest life expectancies in Europe, reaching 82.4 years at birth. This demographic shift represents a profound population transformation that introduces new challenges related to health systems, sustainability, and the maintenance of personal autonomy. Although global populations are aging rapidly, the growing longevity is not necessarily accompanied by longer periods of good health. This situation highlights the need to redefine healthy aging by placing greater emphasis on functional capacity as a central element [2].

Aging is, then, a progressive decline in fitness due to the increasing deleteriome, adjusted by genetic, environmental, and stochastic processes [3]. The biological aging of human organ systems reflects the interplay of age, chronic disease, lifestyle, and genetic risk that exposes the multisystem nature of human aging in health and chronic disease [4]. Thus, aging has been associated with frailty and functional limitations due to three factors: an irreversible biological process; deconditioning due to a sedentary lifestyle; and the effects of comorbidity [5]. With aging, there is a deterioration in the functional reserve that increases the sensitivity to external aggressions that cause frailty, sarcopenia, falls, disability, and hospitalization, with a deterioration in the quality of life and physical condition [6,7]. These decreases have been associated with an increased incidence of type 2 diabetes [8], cardiovascular disease [9], and risk of falls [10]. Moreover, with aging, there is a deterioration of cognitive functions [11,12].

There are numerous modifiable factors which could reduce premature death, prevent morbidity and disability, and improve the quality of life and well-being such as physical activity (PA); higher levels of PA increase the odds of healthy aging by 39% [13] and are related to diminished odds of being in the low-stable or fast-decline groups of healthy aging trajectories [14]. In particular, vigorously active men and women live 6.3 years longer in good health and 2.9 years longer without chronic diseases between the ages of 50 and 75 compared to sedentary persons [15]. In this regard, healthy aging, in a holistic sense, is the process of developing and maintaining the functional capacity that enables well-being in older people [16]. Several studies have shown that moderate PA (MPA) reduces mortality; has a positive effect on the prevention of coronary heart disease; reduces blood pressure; and prevents stroke, type 2 diabetes, and the risk of developing dementia and cognitive impairment, which could prevent falls and improve quality of life [17,18,19,20,21]. In addition, older people with higher physical fitness and higher PA levels show more efficient brain activity and higher executive function [22]. By contrast, greater sedentary time was related to an increased risk of all-cause mortality in older adults [23]. Therefore, older adults may benefit from the joint prescription of undertaking adequate moderate and vigorous PA (MVPA) and avoid prolonged sitting [24]. In order to promote PA in older people, several activities have already been tried such as the following: walking; sport; dancing; callisthenic exercises; games; PA in house/garden; work-related PA; PA for transportation; multi-component exercise interventions; resistance exercise; functional-based training; or circuit training [25,26,27]. In particular, walking activities are a major contributor to PA in older adults [28]. Walking or brisk walking is the main example of moderate-intensity activity recommended by public health guidelines [29,30] and can reduce rates of chronic disease and ameliorate rising health-care costs [31]. In this regard, walking provides several health benefits such as weight loss [32], improves many risk factors for cardiovascular disease [33], survival benefits [34], and improves physical fitness [35] and mental health. Walking ≥2 h/day was especially significantly associated with lower all-cause mortality [36].

Despite all these benefits, worldwide, one in four adults currently do not meet the global PA recommendations established by the World Health Organization (WHO) [37], that adults 65 and older should engage in 150 min of moderate- or 75 min of vigorous-intensity aerobic activity and two or more days of muscle-strengthening activity (i.e., strength/resistance training) per week. In particular, in the Spanish population, Latorre et al. [38] show that from 2009 to 2017, levels of PA and the perception of their health status in Spanish elderly people worsened, and their levels of illness increased, which could be associated with a deterioration in their walking ability. In 2013, the global cost of physical inactivity was estimated at USD 54 billion per year in direct care, with an additional USD 14 billion attributed to low productivity; therefore, physical inactivity accounts for between 1 and 3% of national health-care costs [37]. Consequently, it is necessary to prioritize and expand policies to increase PA levels in this population [39].

Nowadays, the use of daily steps, easily tracked through smartphones and smartwatches, is becoming a measure of PA for the population. This can, in turn, be considered a simple and practical target to stimulate mobility and reduce sedentary behavior, especially in adults and the elderly. Consequently, a daily step count (DSC) has several advantages as a metric for assessing PA: it is intuitive, easy to measure, objective, and represents a fundamental unit of human ambulatory activity [40]. In healthy older people especially, 30 min of daily MVPA accumulated in addition to habitual daily activities is comparable to taking nearly 7000–10,000 steps/day [41]. In this regard, a higher DSC may be an indicator not only of greater MVPA but also of the relationship between light PA and sedentary lifestyle, particularly among those older people who are less physically active [42]. Evidence from longitudinal studies established that walking an additional 1000 steps per day can help lower the risk of all-cause mortality, and reduce the significance of cardiovascular diseases causing morbidity and mortality in adults, and that health benefits still exist below 10,000 steps per day. Other previous studies [41,43] recommend increasing the number that an individual usually takes per day by 2500 steps to obtain positive repercussions on health, and that adults who walk over 12,500 steps are ‘highly active’ [41]. In particular, among older people, taking ≈6000 to 9000 steps per day was related with a 40% to 50% lower risk of cardiovascular disease, compared with taking ≈2000 steps per day [44]. Likewise, taking more steps per day was linked with a gradually lower risk of all-cause mortality, up to a level that differs by age, with the risk plateauing for older adults (aged ≥60 years) at approximately 6000–8000 steps per day [45]. In turn, walking ≥5000 steps/day is a cut-off threshold to recommend for reducing the risk of falling in older people who have a low risk of falling [46]. In terms of walking intensity, in adults the optimal step-rate cut-point is 100 step/min for MPA and 130 step/min for vigorous physical activity (VPA) [47], and cadence thresholds of 100, 110, and 120 steps/min were associated with three, four, and five metabolic equivalents (METs), respectively, in 61–85-year-old adults [48].

On the other hand, aging is marked by substantial differences between people of the same chronological age, which has led to an ongoing search for biomarkers capable of reflecting this biological heterogeneity [49]. Quantitative biomarkers help capture these differences by providing estimates of physiological age, offering insight into the degree of “healthy aging” and, in some cases, informing predictions about future health and longevity. Among phenotypic biomarkers, measures of physical function and anthropometry stand out as the most practical and widely applicable indicators [50]. Assessing physical capacity offers a practical and accessible way to detect signs of accelerated aging and to estimate an individual’s biological age [51]. Performance-based functional tests—such as handgrip strength, chair-rise tasks, gait speed, complex walking assessments, the timed up-and-go test, standing balance measures, and the six-minute walk—are widely employed to monitor biological aging [52,53,54,55]. Additionally, DSC could also represent a biomarker of healthy aging, because walking activities are a major contributor to PA in older adults [28]. However, one of the major limitations of studies that associate DSC with healthy aging is that they only provide data on the number of steps, often without considering the intensity of walking (this can be expressed through speed or heart rate). Moreover, issues related to reverse causality could cast doubt on the health benefits exclusively associated with the number of daily steps. Previous studies showed that a brisk walking speed (>4 miles per hour) was associated with a lower hazard for all-cause, cardiovascular disease, respiratory disease, and chronic obstructive pulmonary disease mortality in both men and women, compared to slow walking speed [56]. Moreover, a higher than usual walking pace is associated with a deceleration of the acceleration of all four classical epigenetic clocks’ acceleration [57]. In this regard, slower epigenetic aging is seen to be related to higher energy expenditure, step counts, and more time spent in MVPA [58], thus highlighting the importance of walking intensity in obtaining health benefits. There is inter-person variability in step intensity, which may misperceive the relation between step count and mortality; therefore, step intensity should be considered when assessing the association between step count and mortality [59]. Therefore, understanding step intensity is essential to interpret the meaning of daily steps in older populations.

Thus, despite these emerging public health benefits of walking steps, there is insufficient evidence to recommend the number of daily steps needed for health promotion and an appropriate translation of public health guidelines in terms of steps/day is still not widely available upon [41]. Therefore, the objective of this study was to assess PA measured by DSC in older adults, analyzing differences across age groups and sex, and highlighting the insufficiency of step count alone as a health indicator due to the lack of intensity measurement. The hypothesis of this study is that DSC is significantly influenced by both age and sex in older adults. However, when age is considered, the explanatory power of DSC for health outcomes diminishes, especially due to its inability to reflect the intensity of PA.

## 2. Materials and Methods

### 2.1. Participants

A cross-sectional study was conducted involving adults aged 60 years and older living at home. Participants were recruited using a non-random convenience sampling strategy. Recruitment was conducted through direct contact with older adult groups and community agents, municipal health and aging programs, university outreach activities, organized physical activity groups for older adults, and local associations of older adults. An a priori sample size calculation was performed using G*Power software (version 3.1.9.7 for windows) [60]. The following parameters were selected for analysis of covariance (ANCOVA): small-to-moderate effect size f = 0.22, α level of 0.05, a power level of 0.90, and 8 groups (2 sexes × 4 age groups), considering 1 covariate. Based on these parameters, the required total sample size was estimated at 385 participants. The final sample of 668 older adults (age = 71.33 ± 8.11 years; age range = 60–100 years), 460 women, exceeded this requirement, providing excellent statistical power for detecting group differences while controlling for potential confounders. The sample was selected from a large region of Spain containing both urban and rural populations. The inclusion criteria were as follows: (a) older than 60 years; (b) independent ambulation; (c) free of any pathological disorder associated with the visual or vestibular systems, orthopedic, or gait difficulties; (d) free of any disease that requires daily drugs that would affect gait performance; (e) non-institutionalized individuals. The study was completed per the norms of the Declaration of Helsinki (2013 version). The study was approved by the Ethics Committee at the University of Jaen (Reference code: OCT.20/7.PRY., 26 October 2020). Demographic variables were categorized as follows: marital status was grouped as married versus unmarried; living alone was defined as residing in a single-person household; smoking status was classified as never smoked versus ever smoked (current, occasional, or former); alcohol consumption as abstainer versus consumer (former, moderate, or heavy); and participation in organized physical activities as yes versus no. Taking into consideration that daily steps are associated with the age of the subjects, the participants were organized into chronological age groups in which this association did not occur. Four age groups were made: 60–68 years; 69–73 years; 74–79 years; and >80 years.

### 2.2. Materials and Testing

Body mass was measured using a weighing scale (Seca 899, Seca, Hamburg, Germany) and body height was assessed with a stadiometer (Seca 222, Seca, Hamburg, Germany). Body mass index (BMI) was calculated by dividing body mass (kg) by body height^2^ (in meters). The BMI was categorized according to World Health Organization (WHO) criteria (<18.5 kg/m^2^, underweight; 18.5–24.9 kg/m^2^, normal weight; 25.0–29.9 kg/m^2^, overweight; and ≥30 kg/m^2^, obese) [61]. Waist circumference (WC) was measured using a Seca^®^ 201 tape measure (Hamburg, Germany) at the height of the umbilical scar. The waist-to-height ratio (WtHR) was obtained by dividing the WC by the height in cm. A ≥ 0.5 cut-off point is related to a higher cardiovascular risk [62]. Also, an OMRON^®^ digital electronic monitor model HEM 7114 (Hoffman Estates, IL, USA) was used to analyze blood pressure.

To analyze leg strength, the 30 s sit-to-stand test (30 s STST) [63] was used. This test involves counting the number of times within 30 s that individuals can rise to a full stand from a seated position with their back straight and feet flat on the floor and without pushing off the chair with their arms. Moreover, 10 repetitions of the sit-to-stand test (10 s STST) as fast as possible were conducted, with the time being registered with a manual stopwatch. To analyze cardiorespiratory fitness, the 6 min walking test (6MWT) was used [64] in which participants had to walk as far as possible (without starting to run) around a rectangular loop of 50 m for 6 min. The total distance walked in the 6MWT was recorded in meters.

Gait performance: Gait speed (GS) (seconds) was measured using two double-light barriers (WITTY; MicrogateSrl, Bolzano, Italy; accuracy of 0.001 s) that were placed at the beginning and at the end of a 10 m corridor. The GS test required participants to walk 10 m as quickly as possible without transitioning into a run. The faster of two attempts was retained for analysis. No explicit start signal was given, allowing each participant to begin the task autonomously.

Depression was assessed using the Geriatric Depression Scale [65] which consists of 15 items with a pattern of dichotomous response (yes or no) investigating cognitive symptoms of a major depressive episode during the past fortnight. The scores are from 0 to 15 points, with a score of 5 or more indicating probable depression. The Spanish version [66] was used.

The quality of life was assessed using the 12-item Short Form health survey (SF-12) [67]. The 12 items of SF-12 may accurately reproduce the two summary component scores, the Physical Component Summary (PCS) score and Mental Health Component Summary (MCS) [68]. Higher scores indicated high quality of life.

Fall-related risk patients were asked to recall how many falls they had in the last three years. Falls were defined as unintentionally coming to rest on the floor or a low surface (bed, chair, etc.). Moreover, generalized pain was assessed using a 10 cm visual analogical scale (VAS) where 0 means ‘no pain’ and 10 ‘a lot of pain’.

Furthermore, an abbreviated version of the Charlson Comorbidity Index (CI) was used, an established index for assessing comorbidities [69].

To assess complex daily functioning and independent living abilities, we administered the Lawton Instrumental Activities of Daily Living (IADL) scale [70]. This tool comprises eight items, with total scores ranging from 0 to 8 in women and from 0 to 5 in men, where higher values indicate greater autonomy.

Executive function, specifically processing speed and cognitive flexibility, was evaluated using the Trail Making Test (TMT) [71]. The test includes two components. In TMT-A, participants connect 25 circled numbers in numerical order as quickly as possible, engaging visuospatial scanning, sustained attention, and basic motor skills. TMT-B follows the same format but requires alternating between numbers and letters in ascending sequence (e.g., 1–A–2–B), which places greater demands on mental flexibility and divided attention [72]. Performance was quantified by completion time, with longer durations reflecting poorer outcomes.

PA was measured by a short version of the short form of the International Physical Activity Questionnaire (IPAQ), Spanish version [73], which is a validated survey that measures the participation in MVPA and sedentary behavior over the past seven days [74]. Among other PA questionnaires, the IPAQ-s showed acceptable to good results for both reliability and validity [75]. It is a valid tool for assessing PA in adults and older adults, and a test and retest would provide an acceptable measure of total SB and MVPA [76,77,78]. When only self-reporting is feasible due to time and resource limitations to analyze a large population, assessment of MVPA with the IPAQ-SF is recommended [79].

Weekly step counts were obtained using the Xiaomi Mi Band 4 (MB4; Xiaomi Corp., Beijing, China), an affordable wrist-worn activity monitor worn on the non-dominant wrist according to the manufacturer’s instructions. The device estimates step volume using integrated three-dimensional accelerometers and a 3-D gyroscope, while heart rate is measured through an optical photoplethysmography sensor. Data were synced via Bluetooth to the Mi Fit mobile application (Huami Co., Ltd., Hefei, China). This wearable has been validated previously in older adults [80]. Additionally, based on previous studies [41,81], a level of approximately 7100 steps/day has been suggested to equal 30 min/week MVPA in older adults, and was considered as a recommendation to promote health in older adults between 60 and 79 years of age. Due to the higher levels of morbidity found in those over 80 years of age, the recommendation of 4600 steps per day was established. Participants in this study were categorized based on whether they were able to reach these values or not.

### 2.3. Procedure

In three separate sessions (48 h apart), a team of researchers previously trained in conducting the different tests evaluated the participants. During the first testing session, the anthropometric variables and physical fitness tests were analyzed. In a second session, the gait tests were performed. In a third session, cognitive measures were recorded. The questionnaires were completed individually and in the presence of the researchers (who respected data confidentiality and clarified any potential doubts or questions). In the same session, an MB4 was placed on the non-dominant wrist of each of the participants, which was worn throughout the 7 days a week, 24 h a day. Participants whose consecutive 7-day step counts were unavailable and who had unreliable daily step counts (DSCs) (<500) were excluded [82]. Data collection was carried out from 2022 to 2024 and throughout the four seasons of the year.

### 2.4. Statistical Analysis

The statistical analyses were performed using SPSS version 22.0 (SPSS Inc., Chicago, IL, USA). The significance threshold was set at α = 0.05. Descriptive results are reported as means, standard deviations, and percentages. Prior to the main analyses, normality and homogeneity of variances were examined with the Kolmogorov–Smirnov and Levene tests. Differences between sex and age groups were evaluated using analysis of covariance (ANCOVA) adjusted for age, sex, and the Charlson Index, with Bonferroni corrections applied to post hoc comparisons. Non-parametric data were analyzed using the Mann–Whitney U test or the Kruskal–Wallis test when appropriate. The chi-squared test was used to compare nominal variables between groups. Associations between DSC and health-related outcomes were examined using partial correlations adjusted for age and sex. The magnitude of correlation among measurement variables was set according to Hopkins et al. [83]. To identify independent sociodemographic and behavioral determinants of DSC, a multiple linear regression model was performed. Finally, the coefficient of variation (CV, %), given as a percentage SD/mean × 100, was calculated as a measure of DSC variability.

## 3. Results

In this study, 59.1% of women vs. 88.0% of men were married (*p* < 0.001), 71.8% of women vs. 92.3% of men lived alone (*p* < 0.001), 76.7% of men vs. 48.7% of women declared never having smoked (*p* < 0.001), and 57.6% of women vs. 20.8% of men declared they were non-consumers of alcohol (*p* < 0.001). Regarding participation in organized physical activities, 58.1% of men vs. 40.2% of women do not participate in any (*p* < 0.001). Without significant differences between the sexes (*p* ≥ 0.05), in the total sample, a prevalence of overweight and obesity of 47.5% and 24.4%, respectively, was found. Table 1 shows the different health outcomes analyzed in relation to the sex of the participants. Women significantly show a lower number of steps than men; these differences were only significant in the 69–73 years age group (*p* = 0.009). Moreover, women show worse health outcomes than men in various variables, such as self-perceived health status, pain, levels of depression, number of falls, and executive functioning.

**Figure 1:**
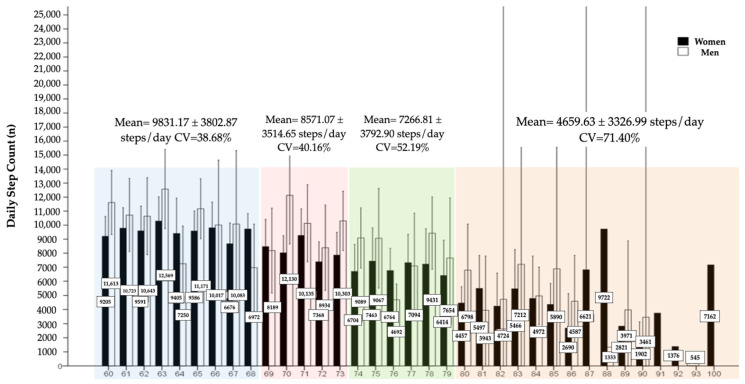
Evolution of daily steps across age groups. Different colors indicate different age groups.

Regarding total participants, men = 69.2% vs. women = 60.0%; *p* = 0.022 meets with the recommended reference values of DSC. With regard to the age groups according to sex, a significant reduction in DSC is observed in both men and women, as well as an increase in CV of DSC (Figure 1).

Table 2 shows different health outcomes analyzed in relation to the age group, distinguished by whether or not they meet the DSC reference values. It can be observed that, in the total sample, the group that meets the reference values demonstrates better results in various health variables compared to those who do not, including BMI, cardiometabolic risk, perceived health, depression, morbidity pain, and executive functioning, although physical fitness levels are not affected. In the different age groups, where age and sex are controlled, many of these differences disappear, with significant differences observed only in certain groups and specific variables, such as perceived health. These results are corroborated in the correlation analysis shown in Table 3, where modest or low correlations between DSC and various health outcomes are observed across age groups. It is worth noting that regardless of the age group, in the group of people who meet the recommended DSC, there is a significantly higher percentage of individuals who engage in VPA compared to the group that does not meet the recommended values.

**Figure 2:**
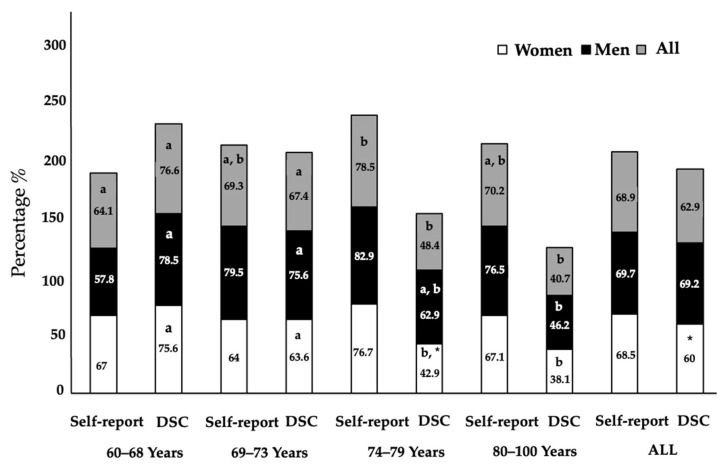
Evolution across age groups related to sex of the percentage of compliance with the recommended DSC reference values and self-report PA; Different subscript letters indicate significant differences (*p* < 0.05) within group. * Indicate significant differences (*p* < 0.05) between sexes; DSC: Daily Step Count.

Figure 2 shows the percentage of compliance with PA recommendations based on both self-reported PA and DSC. No significant differences (self-reported PA vs. DSC) are found in any age group, either in the total sample or by sex. Comparing the different age groups, in the total sample and among women, there is a significant reduction (*p* < 0.001) in compliance with DSC recommendations starting at age 74, and among men starting at age 80. In the total sample, significant differences (*p* = 0.022) are found in DSC compliance between the sexes, with men showing a higher percentage of compliance. These differences become significant (*p* = 0.044) only in the 74–79 age group. Considering compliance with PA recommendations based on self-reported data, significant differences are observed between age groups in the total sample and among men (*p* = 0.039 and *p* = 0.010, respectively), but this does not reflect a reduction in the compliance percentage with age.

**Figure 3:**
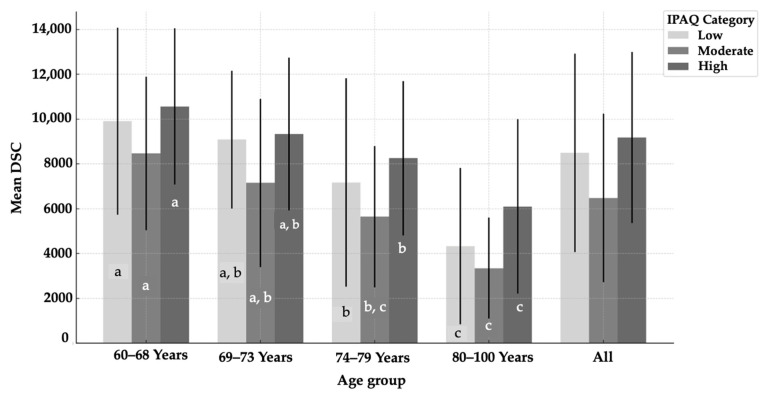
Daily Step Count related to PA levels by age groups. Different subscript letters indicate significant differences (*p* < 0.05) in within-group PA levels by age groups; DSC: Daily Step Count.

Figure 3 illustrates the distribution of mean DSC across four age groups and three levels of physical activity (IPAQ: low, moderate, high PA). DSC differed significantly across IPAQ physical activity levels in several age groups when adjusting for sex. In the 60–68 years group, individuals classified as highly physically active showed significantly higher DSCs compared to those with moderate physical activity (*p* = 0.001). Similar differences between high and moderate IPAQ levels were also observed in the 74–79 years (*p* = 0.006) and 80–100 years groups (*p* = 0.003). These findings suggest that the distinction between moderate and high PA levels is particularly relevant in influencing DSC, especially among younger and older adults, after controlling for sex. At all levels of PA, there is a significant reduction (*p* < 0.05) in DSC across age groups.

A multiple linear regression analysis was performed to identify sociodemographic and lifestyle predictors of DSC. The model was statistically significant (F(17, 472) = 17.09, *p* < 0.001), explaining 38.1% of the variance in DSC (R^2^ = 0.381; adjusted R^2^ = 0.359). Age was a strong negative predictor: for each additional year of age, participants accumulated 224 fewer steps per day (B = −224.1, *p* < 0.001). Sex had a significant effect, with men walking 805 more steps per day than women (B = +805.4, *p* = 0.030). Participants who engaged in organized physical activity walked 909 more steps per day than those who did not (B = +909.3, *p* = 0.004). Individuals in the high physical activity category (IPAQ level 3) accumulated 1517 more steps per day than those in the low category (B = +1516.6, *p* < 0.001). Additionally, participants who had smoked walked 2702 fewer steps per day compared to those who had never smoked (B = −2702.2, *p* = 0.001). Conversely, those who reported alcohol consumption walked 1086 more steps per day than abstainers (B = +1085.9, *p* = 0.001). The lower step counts observed among non-drinkers may be partially explained by the high proportion of women in this group. Other variables, including marital status, moderate IPAQ level, and weight status, did not show significant associations with daily step count.

Finally, Table 3 shows the correlation analysis between DSC and various health variables. In the total sample, several significant correlations are observed, modest or low, correlations that in some cases disappear in the different age groups. Additionally, DSC was inversely associated with age (r = −0.460, *p* < 0.001, R^2^ = 0.212, Y = 24,903,93 − 234.36X), in both men (r = −0.450, *p* < 0.001) and women (r = −0.468, *p* < 0.001).

## 4. Discussion

The objective of this study was to assess the PA level measured by DSC in older adults, analyzing differences across age groups and sex and highlighting the insufficiency of step count alone as a health indicator due to the lack of intensity measurement. The findings of the current study indicate that although step volume differs clearly by age and sex, its relationship with health outcomes is generally weak once these factors are accounted for. Four key results emerged: (1) daily steps decline markedly across age groups and show substantial variability between individuals; (2) women accumulate fewer steps and display less favorable health profiles than men in depressive symptoms, number of falls, pain perception, and executive function measures; (3) meeting age-specific step recommendations is associated with better anthropometric, psychosocial, and cardiometabolic markers, but many of these differences disappear after adjusting for age and sex; and (4) correlations between step count and physical, cognitive, and functional outcomes are modest and often inconsistent across age strata. Taken together, these findings underscore that DSC alone offers a limited and incomplete representation of health status in older adults.

Beyond step volume, the analyses of the current study highlight the importance of activity intensity for understanding health in older adults. In this regard, older adults who meet the recommended DSC present a more favorable profile across multiple health indicators and report higher levels of vigorous PA according to the IPAQ. This suggests that step accumulation is indirectly linked to higher activity intensity and broader behavioral patterns associated with healthier lifestyles. Nevertheless, it is important to note that, in the total sample, the group that meets the step recommendations is also younger, and this age difference explains part of the observed health advantage. When comparisons are made within age strata, many of these differences become attenuated, underscoring the strong confounding role of age. The addition of mean weekly heart rate provided only limited incremental explanatory value, suggesting that older adults tend to accumulate the majority of their daily steps at low walking speeds and physiological loads. Consequently, the physiological impact of their habitual ambulation is relatively small, which may partly explain the weak predictive capacity of step count. These findings collectively indicate that step volume captures only a narrow aspect of habitual activity and should be interpreted alongside intensity-related metrics when evaluating health status in aging populations. Therefore, meeting step recommendations reflects a combination of age-related mobility patterns and indirectly higher activity intensity, rather than step count itself being a strong independent predictor of health in older adults. Overall, our findings demonstrate that daily step count is an insufficient standalone indicator of health in older adults because it largely reflects low-intensity ambulation and age-related mobility patterns rather than meaningful physiological load. Only when activity intensity is considered—either through self-reported IPAQ categories or objective heart rate measures—does the interpretation of daily activity begin to align more closely with relevant health outcomes.

Taking into account the sociodemographic variables as a whole, daily step count is strongly influenced by behavioral and sociodemographic factors, particularly self-reported activity intensity (IPAQ level) and participation in structured physical activity. These two variables alone accounted for increases of 900–1500 steps/day, highlighting that step accumulation reflects broader activity patterns beyond simple ambulation. Interestingly, individuals who reported alcohol consumption walked more than abstainers, although this finding may be influenced by confounding, given that the non-drinking group included a disproportionately high percentage of women, who also showed lower step counts on average. Similarly, smokers walked fewer steps than those who never smoked, which may reflect underlying health conditions or reverse causality rather than a direct effect of smoking history. Moreover, age and sex also played meaningful roles, with older participants and women accumulating fewer steps. In contrast, education, marital status, and weight status did not independently contribute once other variables were included. These findings further reinforce the central conclusion of this study: daily step count is shaped by the demographic and behavioral context, and therefore cannot be interpreted as an isolated indicator of health without considering activity intensity and lifestyle factors.

In both men and women, the data obtained in this study regarding DSC are generally not consistent with the expected values previously published for older adults. The differences between studies may be due to the use of different devices and monitoring periods. In the current study, DSC was higher than in previous studies [42,46,84,85,86]. For example, Şahin et al. [84] conducted a study with a population aged 65 to 83 years who each wore a pedometer for a week, but it was removed during personal care activities, such as sleeping, showering, or bathing. The average values they obtained were 6011 ± 2089 steps. Similarly, with a sample of older adults (mean age, 74.3 years), Amagasa et al. [42] recorded daily steps with an accelerometer over the course of a week, excluding sleep time and aquatic activities, obtaining average values of 5.412 ± 2878 steps.

Moreover, the data from the present study confirm the well-known sex- and age-related differences in steps/day in older adults [85,87,88,89] with men showing a higher number of steps than women, and older individuals taking the fewest daily steps. This sex and age difference in DSC could be related to daily PA levels in which previous studies have shown the decrease in women’s PA levels with age in comparison to men [90,91]. However, in the present study, no significant differences between men and women were found in PA levels. Yet, in accordance with other previous studies, women deteriorated more than men in relation to quality of life, pain, depression, number of falls, and executive function [90,92,93]. This could explain the differences in physical functioning between women and men where older women have more limitations than older men [94] and women’s self-perception regarding mobility is a subjective measure [95].

Focusing on meeting the recommended reference values of DSC, in the present study significant differences were found between men (69.2%) and women (60%), and the compliance of reference values decreases when age increases. In men, this non-compliance occurs from the age of 80, and in women, it occurs five years earlier, starting at the age of 74. However, according to the study by Dohrn et al. [81], a wide variation was found in DSC within all age groups, indicating that high levels of PA may be sustained even among the oldest—for example, one 100-year-old participant took more than 7000 steps daily. Moreover, in all of the samples, it was also found that the prevalence of meeting PA recommendations was similar between device-measured (62.9%) and by self-report (68.9%). Similarly to a previous study [81], age differences were much more pronounced with objective measures than by self-report. This study showed that the prevalence of meeting PA recommendations was 59% device-measured and 88% by self-report. Likewise, in accordance with Amagasa et al. [42], DSC was associated with MVPA, and age groups who met DSC recommendations during the day also accumulated less time spent in LPA and SB and more in MVPA. Therefore, higher DSC can be an indicator of not only a larger relative contribution of time spent in MVPA, but also a higher ratio between LPA and SB, particularly among those who are the least physically active [42]. Therefore, DSC can be useful to identify older adults with unhealthier movement behavior, that is, high SB and low MVPA [96]. However, analyzing the relationship between DSC and PA levels, although a previous study indicates that DSC was positively associated with MVPA and negatively with the time spent in light PA and sedentary time [42], in the current study there were no significant differences in DSC between participants who spent more time in light activities and those with higher levels of MVPA. The methods used to characterize PA levels, questionnaires versus accelerometery, could possibly explain these discrepancies. In addition, the self-perception of PA levels reported by the IPAQ overestimated the level of PA [97].

In the current study, both men and women who meet the reference values demonstrate better results in various health variables compared to the group that does not meet the reference values such as bodyweight, cardiometabolic risk, perceived health, depression, pain, and EF. However, overall, the results do not show a clear and positive dose–response trend, where, as an individual takes more daily steps, various aspects of their functionality and health improve, due to age being a conditioning factor in these relationships. It only seems evident that anthropometric markers of overweight and obesity decrease when subjects take more daily steps. In this regard, significant weak correlations were found between daily steps and BMI or cardiometabolic risk parameters. Previous studies confirm a modest to low inverse correlation between daily steps and BMI [84,86,98] and waist circumference [98] and a positive, moderate correlation with self-perceived personal health, but no significant association is found with weekly PA levels. Likewise, another study indicates that daily step volume and intensity were inversely associated with cardiometabolic risk in community-dwelling older adults [99]. In this regard, these associations may be due to the fact that the metabolic cost of walking is higher in older adults than in younger adults [100], which may have positive effects on weight status.

On the other hand, although high levels of PA are associated with better physical fitness and health outcomes [101], especially with muscle strength and muscle power in older adults [102], in the present study and in line with previous work [84], no significant associations were found between daily steps and various physical fitness variables in the total sample, except in the 74 to 79 age group, where leg strength is associated with the DSC. Likewise, in another study, after controlling data for age and/or sex, lower-extremity function (walking speeds and knee extension torque) showed significant positive relationships with the DSC, especially in individuals ≥75 years of age [103]. In addition, previous studies show modest correlations between DSC and cardiorespiratory fitness and leg strength [104,105]. Another study highlights that in adults ≥75 years, total steps walked was associated with better gait speed [106].

Moreover, in certain self-reported psychosocial variables, such as perceived health, depression, pain, and executive functions, DSC shows modest to weak associations. In the current study, regardless of age groups, there were significant associations between DSC and health-related quality of life. Another previous study reported similar findings [107]. In relation to depressive symptoms, most patients with moderate to severe depression do not achieve sufficient PA [108]; the findings of the current study are consistent with those of Tudor-Locke et al. [109] who showed that accumulating ≥7000 steps a day could provide the greatest protection against depressive symptoms in older people [110]. Likewise, a previous study indicated that a higher number of chronic musculoskeletal pain sites is associated with a lower step count in community-dwelling older adults [111]. Finally, the present findings seem to be consistent with other research which found a significant relationship between average daily steps and executive functioning [112]. In particular, 7000 steps/day was related to an approximate reduction of 60% in the rate of subjective cognitive decline [113].

Regardless of the daily step volume, the majority of DSCs taken by older people are at a low intensity [31,114] and most older adults did not achieve the intensity of ≥6 METs [48]. Because the relative energy cost of walking and other daily activities is higher in older adults than younger adults, the observed benefits of DSC may vary depending on the interaction between step intensity and age. Hence, a single recommendation for step counts may not be appropriate for all adults [114]. In addition, there is inter-person variability in step intensity, which may confound the association between step count and mortality [59]. Among older adults, both a high step volume and step intensity were significantly associated with lower hospitalization and all-cause mortality risk [115]. Increasing the step volume and intensity may benefit older people. A higher step intensity may provide additional benefits [114], although intensity is a main driver of reduced mortality risk, suggesting that the intensity of PA rather than the quantity matters for longevity [116].

In the present study, when the results are adjusted for the age and sex of the participants, DSC shows a smaller effect on the different health outcomes of the participants. Furthermore, it seems that MVPA is actually the variable that could better explain the modest positive effects on the different health outcomes of this population, as the age groups that meet the DSC recommendations exhibit a higher percentage of MVPA. Additionally, across all age groups, there are no significant differences in DSC between those who engage in LPA and MVPA.

Although there is increasing evidence with regard to the effects of DSC on health outcomes and the reduction in mortality risk in different populations [31,114,117,118,119,120], many of these studies have not been able to resolve the problem of reverse causality (diseases that cause a low DSC), which is, in our opinion, their greatest limitation. In addition, data are needed to identify a specific minimum threshold of DSC needed to obtain an overall health benefit [119]. In addition, methodological inconsistencies are observed in the different studies, due to the recording device used (accelerometer vs. pedometer) or the recording time (between 2 and 7 days, ≥10 h/day) [119] and much data come from observational studies; therefore, causal inferences cannot be made [45]. Moreover, DSCs were investigated only at the baseline, but PA behavior may change over time and is influenced by various factors (e.g., age, sex, socio-economic status, disease state) [121]. In addition, DSC measured by the accelerometer may be due to PA other than walking (e.g., dancing, gardening, housework) and the device does not detect non-ambulatory activities (e.g., swimming, cycling) [122]. Many of the previous studies do not take into account walking intensity [82,119,123,124,125], and others express it in relation to cadence, rather than walking speed, which is based on both cadence and step length; older adults reduce walking speed primarily at the expense of step length [126,127]. Sometimes studies even used incorrect calculations of cadence or were imprecise [31,122] or the calculations were made under laboratory conditions [48], which compromises their ecological validity. In addition, inter-individual variation in step rate was apparent due to anthropometric differences such as height and leg length [128,129]. Although a higher step rate consumes more energy expenditure, there is no significant relationship between aerobic capacity and step rate within each speed [130].

Therefore, a variety of health benefits related to PA depends upon the frequency, intensity, duration, and type of exercise. The intensity of exercise refers to the rate of metabolic energy demand during exercise, and has important implications for the health benefits elicited by regular PA, because adaptations to regular exercise will be obtained only when sufficient disturbance to homeostasis has occurred [131] and for this, it is necessary to overcome a certain degree of intensity of exercise. According to the overload principle of training, exercise below a minimum intensity, or threshold, will not challenge the body sufficiently to result in increased VO_2_max and improvements in other physiological parameters [132]. The minimum threshold of intensity for benefit seems to vary depending on an individual’s current cardiorespiratory fitness (CRF) level and other factors such as age, health status, physiologic differences, genetics, habitual PA, and social and psychological factors [132]. The following are recommendations for PA in older people: 12–14 on the Borg Scale (55–70% heart rate reserve or maximum exercise capacity) or power training at 40–60% of 1RM [30]. Traditionally, heart rate was the most popular method of aerobic forms of exercise in terms of exercise intensity prescription. According to the recommendations, the intensity in aerobic exercise should be 12–14 on the Borg Scale (55–70% heart rate reserve or maximum exercise capacity) [30]. In this study, the average daily heart rate was significantly higher in the group that met the DSC reference values. In this regard, and in relation to the intensity associated with DSC, the results of the current study indicate that DSC alone is not a robust indicator of overall health in older adults. This is because most steps accumulated in this population occur at very low walking speeds and intensities, providing limited physiological stimuli and therefore weak associations with key health outcomes. In contrast, IPAQ-derived intensity levels showed clearer and stronger relationships with functional capacity, physical health, and comorbidity, highlighting that intensity rather than volume is the primary driver of health-relevant adaptations. Although we also included heart rate as an objective marker of physiological intensity, its contribution to model performance was small, improving explained variance only modestly. This suggests that HR captures some additional physiological load, but not enough to compensate for the inherently low-intensity nature of most daily ambulation in older adults. Taken together, these findings support our conclusion that step count alone is insufficient and that the meaningful interpretation of PA in older adults requires a consideration of activity intensity, while acknowledging that heart rate adds only limited incremental value in this context.

Hence, walking is the simplest, cheapest, most universal resource and from a public health perspective it is recommended to older people to promote their health. Increasingly, walking is becoming the simplest, most versatile and adaptable form of PA for any person and environment, without the need of a professional—particularly, as occurs in many countries, where the depopulation of rural areas is increasing and there are no adequate services that allow a healthy PA program for older people. Increasing the time spent stepping should contribute both to reducing sedentary behaviors and increasing both light-intensity PA and MVPA [42]. Accordingly, public health policies should promote active locomotion. In this regard, providing the social dimension of participating in leisure walking with peers could significantly increase older adults’ motivation to engage in leisure walking [133].

This study has several limitations. First, the cross-sectional nature of this study did not allow us to determine cause-and-effect relationships between DSC and the rest of the variables analyzed. Moreover, not all human movement is represented by a measure of daily steps taken. In addition, certain age groups are less represented, especially by men, which could affect the characterization of the daily steps of these subjects. A further limitation is the possibility of reverse causation. Because the study is cross-sectional, we cannot determine whether lower step counts contribute to poorer health or whether individuals with worse health simply walk less due to physical or functional constraints. This limitation is inherent to the design and cannot be fully controlled. Finally, other hematological and immunological blood parameters (uric acid, malnutrition biomarkers, etc.) should be considered in future studies.

Nevertheless, this study has several strengths, since the sample comprises many older people from a large region, including rural and non-rural areas, and therefore, to the best of our knowledge, this is the first study with these characteristics carried out in a Spanish population that provides step-based metrics for Spanish older adult people, which can facilitate future training programs, research, and data interpretation. Moreover, the device and protocol used allowed us to collect 24 h of accelerometery data to reduce the chance of missing periods of ambulatory activity. Another important strength is the population-based study sample, which included 123 participants over 80 years.

## 5. Conclusions

In conclusion, the current study warns that DSC could be an insufficient value to evaluate health in older adults in relation to locomotion aspects. Evidently, there are differences between sex and age related to DSC and the general sample of older adults who met the reference values obtained better performance in quality of life, BMI, cardiometabolic risk, depression, and pain. However, no relevant differences were found among these variables in specific age groups. Therefore, it is essential to complement the study of DSC with that of walking intensity to adequately determine its effects on the health of older people.

## Figures and Tables

**Table 1:** Different health outcomes analyzed in relation to the sex of the participants.

	Women Mean (SD)	Men Mean (SD)	*p*-Value
Age (years)	71.42 (7.98)	71.11 (8.39)	0.514
Body mass (kg)	68.20 (10.62)	79.73 (12.11)	<0.001
Height (m)	1.57 (0.06)	1.70 (0.07)	<0.001
WC (cm)	96.13 (13.61)	103.24 (15.69)	<0.001
WtHR (cm)	0.616 (0.08)	0.610 (0.09)	0.707
BMI (kg/m^2^)	27.48 (4.21)	27.63 (4.00)	0.674
Weight status (low weight, normal weight/overweight/obesity, %)	0.4/29.3/45.9/24.3	0.0/24.5/51.0/24.5	0.409
Systolic pressure (mmHg)	127.82 (16.30)	129.39 (17.71)	0.320
Diastolic pressure (mmHg)	76.14 (10.63)	76.68 (11.39)	0.909
Daytime heart rate (bpm)	79.48 (5.87)	78.05 (5.62)	0.002
STST 10 (s)	16.95 (4.52)	15.98 (4.35)	0.143
STST 30 (rep.)	18.98 (5.55)	20.46 (5.72)	0.079
Fast speed 10 m (m/s)	1.87 (0.36)	1.96 (0.31)	0.102
6MWT (m)	536.66 (82.16)	554.51 (75.72)	0.125
PA level (low, mediun, high, %)	31.5/25.1/43.4	30.3/21.2/48.5	0.425
TMTA (s)	83.20 (53.39)	74.43 (57.15)	0.002
TMTB (s)	177.67 (111.35)	154.76 (104.63)	0.005
MCS (0–100)	50.07 (9.28)	53.80 (7.44)	<0.001
PCS (0–100)	45.58 (9.82)	48.00 (8.50)	0.010
Disease or chronic/long-lasting health problem (≥6 month, %)	38.4	32.7	0.212
Geriatric depression (0–15)	2.85 (2.80)	2.04 (2.12)	0.001
People with depression (%)	22.8	11.4	0.001
Lawton Index (0–8)	7.59 (1.06)	6.73 (1.58)	<0.001
Charlson Index (0–10)	3.43 (1.51)	3.46 (1.62)	0.882
10-year survival (%)	63.76 (29.40)	63.54 (30.60)	0.707
Falls (numbers of falls)	1.44 (2.59)	0.84 (1.60)	0.005
Widespread pain (0–10)	3.04 (2.07)	2.21 (1.99)	<0.001
DSC (step count)	7855.46 (3932.94)	8919.08 (4455.65)	0.002

WC = Waist circumference; WtHR = Waist-to-height ratio; BMI = Body mass index; STST = Sit-to-stand test; 6MWT = 6 min walking test; TMT = Trail Making Test; MCS = Mental Health Component Summary; PCS = Physical Component Summary; DSC = Daily steps count; SD = Standard deviation.

**Table 2:** Different health outcomes analyzed in relation to the age group, distinguished by whether or not they meet the daily step reference values.

	All			Age Group: 60–68 Years			Age Group: 69–73 Years			Age Group: 74–79 Years			Age Group: 80–100 Years		
	Do Not Meet RV	Meet RV	*p*-Value	Do Not Meet RV	Meet RV	*p*-Value	Do Not Meet RV	Meet RV	*p*-Value	Do Not Meet RV	Meet RV	*p*-Value	Do Not Meet RV	Meet RV	*p*-Value
Age (years)	74.59 (8.32)	69.41 (7.34)	<0.001	64.25 (2.82)	63.74 (2.69)	0.203	71.28 (1.43)	71.01 (1.48)	0.337	76.04 (1.58)	76.16 (1.71)	0.789	84.69 (3.45)	83.62 (3.45)	0.052
% Men vs. women	25.8/74.2	34.3/65.7	0.027	29.4/70.6	32.9/67.1	0.697	23.8/76.2	35.6/64.4	0.250	20/80	36.1/63.9	0.069	28.8/71.2	36/64	0.516
Body mass (kg)	73.08 (13.03)	71.04 (11.83)	0.083	74.96 (12.79)	71.57 (12.15)	0.039	74.85 (18.05)	71.04 (12.91)	0.173	74.16 (10.93)	71.18 (9.38)	0.107	69.49 (10.94)	68.52 (11.13)	0.633
Height (m)	1.60 (0.09)	1.62 (0.09)	0.027	1.64 (0.08)	1.63 (0.09)	0.750	1.59 (0.08)	1.61 (0.09)	0.167	1.60 (0.08)	1.61 (0.09)	0.500	1.58 (0.09)	1.59 (0.08)	0.604
Waist circumference (cm)	101.82 (17.11)	96.47 (12.76)	<0.001	101.82 (14.75)	95.43 (13.67)	0.004	102.85 (23.87)	97.58 (11.52)	0.649	103.76 (11.56)	98.27 (10.64)	0.027	99.05 (19.26)	98.31 (11.85)	0.845
BMI (kg/m^2^)	28.40 (4.55)	27.07 (3.79)	<0.001	27.93 (4.50)	26.82 (3.75)	0.042	29.56 (6.35)	27.05 (3.71)	0.005	28.90 (3.94)	27.65 (3.38)	0.062	27.78 (3.74)	27.05 (4.56)	0.336
Weight status (%, low weight, normal weight/overweight/obesity)	0.0/22.2/47.6/30.2	0.5/31.2/47.3/21.0	0.010	0.0/32.4/35.2/32.4	0.5/33.8/45.4/20.3	0.180	0.0/23.8/35.7/40.5	0.0/26.4/52.9/20.7	0.052	12.7/55.6/31.7	21.3/55.7/23.0	0.328	0.0, 19.4/59.8/20.8	2.0/39.2/37.2/21.6	0.035
WtHR	0.63 (0.10)	0.60 (0.08)	0.001	0.62 (0.07)	0.60 (0.08)	0.077	0.65 (0.14)	0.60 (0.06)	0.226	0.65 (0.08)	0.62 (0.06)	0.055	0.62 (0.11)	0.62 (0.08)	0.845
Systolic pressure (mmHg)	130.02 (15.55)	127.26 (17.34)	0.276	128.58 (16.73)	125.54 (16.75)	0.435	132.09 (14.63)	128.57 (17.48)	0.647	129.94 (16.22)	131.53 (16.01)	0.458	130.73 (14.54)	130.52 (21.60)	0.918
Diastolic pressure (mmHg)	74.61 (10.69)	77.36 (10.83)	0.006	74.22 (9.52)	77.65 (10.48)	0.044	77.22 (11.00)	75.97 (8.65)	0.904	77.27 (12.19)	78.61 (15.87)	0.789	71.48 (10.09)	76.42 (9.96)	0.086
Daytime heart rate (bpm)	77.59 (5.87)	79.87 (5.64)	0.002	80.03 (5.01)	81.06 (5.47)	0.206	76.95 (5.70)	78.92 (5.75)	0.069	77.02 (5.87)	78.97 (4.60)	0.057	76.25 (6.15)	77.08 (6.11)	0.442
STST10 (s)	16.29 (4.15)	16.84 (4.66)	0.392	16.05 (4.29)	17.16 (4.85)	0.392	15.16 (4.32)	17.53 (4.70)	0.117	17.92 (4.20)	15.97 (3.93)	0.177	15.89 (3.44)	14.45 (3.51)	0.170
STST30 (rep.)	19.95 (5.78)	19.17 (5.55)	0.308	20.76 (5.87)	19.08 (4.99)	0.308	21.47 (6.14)	17.42 (6.03)	0.018	16.65 (4.63)	20.52 (5.64)	0.045	21.05 (5.67)	21.76 (7.04)	0.902
Fast speed 10 m (m/s)	1.93 (0.36)	1.89 (0.34)	0.429	1.98 (0.37)	1.92 (0.33)	0.424	1.99 (0.33)	1.81 (0.36)	0.104	1.87 (0.34)	1.79 (0.32)	0.485	1.85 (0.38)	1.98 (0.29)	0.324
6MWT (m)	544.05 (85.58)	541.31 (77.71)	0.808	545.00 (69.80)	544.46 (81.58)	0.952	551.17 (89.52)	533.27 (71.61)	0.460	533.75 (93.04)	534.70 (64.81)	0.972	546.76 (100.26)	547.69 (87.01)	0.979
IPAQ level: low, mediun, high (%)	32.3/36.7/31.0	30.5/16.5/53.0	<0.001	39.1/34.3/26.6	35.0/17.1/47.9	0.002	29.7/35.2/35.1	31.2/15.6/53.2	0.047	27.1/35.6/37.3	16.7/11.6/71.7	0.001	33.3/41.3/25.4	24.4/22.0/53.6	0.012
TMT (s)	95.26 (63.26)	71.90 (46.66)	<0.001	63.89 (29.87)	55.31 (33.09)	0.007	84.00 (48.19)	77.65 (44.65)	0.322	89.83 (38.07)	92.12 (60.81)	0.322	140.47 (89.84)	106.65 (47.76)	0.163
TMT (s)	193.43 (112.73)	157.93 (106.11)	<0.001	137.55 (67.19)	117.51 (70.01)	0.012	177.29 (102.14)	175.46 (120.39)	0.871	201.34 (116.39)	203.11 (113.87)	0.871	257.33 (123.27)	241.06 (120.04)	0.542
MCS (0–100)	50.17 (9.39)	51.87 (8.57)	0.027	47.84 (10.16)	50.59 (8.36)	0.046	49.54 (10.26)	54.04 (7.55)	0.022	51.41 (8.28)	52.87 (8.52)	0.337	51.51 (8.81)	53.18 (10.34)	0.386
PCS (0–100)	42.59 (10.21)	48.49 (8.33)	<0.001	44.53 (10.21)	48.97 (8.33)	0.001	43.96 (9.82)	48.71 (8.01)	0.014	43.55 (9.95)	48.93 (7.35)	0.004	38.82 (10.07)	45.24 (9.57)	0.001
Geriatric depression (0–15)	3.18 (2.64)	2.30 (2.62)	<0.001	3.00 (2.52)	2.23 (2.77)	0.002	3.61 (2.75)	2.02 (2.30)	0.002	2.79 (2.08)	2.39 (2.23)	0.210	3.45 (3.09)	2.71 (2.56)	0.596
People with depression (%)	25.2	15.8	0.005	22.6	16.3	0.254	38.2	12.3	0.002	20.4	16.1	0.559	24.6	19.0	0.507
Lawton Index (0–8)	7.05 (1.68)	7.49 (1.01)	0.008	7.32 (1.49)	7.67 (0.77)	0.237	7.41 (1.23)	7.40 (0.94)	0.600	7.38 (1.13)	7.32 (1.05)	0.306	6.38 (2.09)	6.90 (1.56)	0.206
Charlson Index (0–10)	4.02 (1.71)	3.10 (1.33)	<0.001	2.97 (1.45)	2.52 (0.99)	0.018	3.67 (1.62)	3.35 (1.03)	0.630	4.04 (1.27)	3.83 (1.08)	0.415	5.25 (1.59)	4.77 (1.61)	0.109
10-year survival (%)	53.13 (32.05)	70.02 (26.37)	<0.001	72.83 (27.62)	81.18 (18.81)	0.148	61.04 (31.38)	65.74 (24.91)	0.875	52.33 (28.24)	57.47 (25.93)	0.396	29.83 (24.16)	35.34 (25.63)	0.229
Falls (numbers of falls)	1.49 (2.64)	1.15 (2.19)	0.085	1.63 (3.57)	1.14 (2.18)	0.409	1.92 (3.20)	1.38 (2.81)	0.209	1.35 (2.20)	0.92 (1.57)	0.458	1.20 (1.45)	0.96 (1.34)	0.038
Widespread pain (0–10)	3.35 (2.19)	2.46 (1.95)	<0.001	2.84 (1.93)	2.37 (1.89)	0.081	3.29 (2.27)	2.43 (1.94)	0.040	3.24 (1.63)	2.45 (1.93)	0.023	3.98 (2.66)	3.00 (2.23)	0.471
DSC (step count)	4142.42 (1849.00)	10,556.38 (3131.86)	<0.001	5261.73 (1447.17)	11,230.82 (3143.92)	<0.001	4953.49 (1794.66)	10,584.38 (2531.65)	<0.001	4252.60 (1481.71)	10,449.21 (2771.58)	<0.001	2487.75 (1291.26)	7725.81 (2873.74)	<0.001

WtHR = Waist-to-height ratio; BMI = Body mass index; bpm: Beats per minute; STST10 = Sit-to-stand test (10 repetitions); STST30 = Sit-to-stand test (30 s); 6MWT = 6 min walking test; TMT = Trail Making Test; MCS = Mental Health Component Summary; PCS = Physical Component Summary; DSC = Daily steps count.

**Table 3:** Correlation analysis between daily steps and various health variables.

	Total r, *p*-Value	60–68 years r, *p*-Value	69–73 years r, *p*-Value	74–79 years r, *p*-Value	80–100 years r, *p*-Value
DSC vs. BMI	−0.168, *p* < 0.001		−0.225, *p* = 0.010	−0.225, *p* = 0.012	−0.200, *p* = 0.026
DSC vs. WC	−0.164, *p* < 0.001	−0.139, *p* = 0.026		−0.252, *p* = 0.017	
DSC vs. WtHR	−0.163, *p* < 0.001			−0.223, *p* = 0.036	
DSC vs. DHR	0.111, *p* = 0.006			0.182, *p* = 0.048	
DSC vs. PCS	0.356, *p* < 0.001	0.285, *p* < 0.001	0.220, *p* = 0.023	0.232, *p* = 0.015	0.342, *p* < 0.001
DSC vs. MCS			0.301, *p* = 0.002		0.239, *p* = 0.016
DSC vs. Depression	−0.256, *p* < 0.001	−0.204, *p* = 0.001	−0.290, *p* = 0.002		−0.229, *p* = 0.020
DSC vs. Survival rate	0.391, *p* < 0.001				
DSC vs. Body pain	−0.257, *p* < 0.001	−0.195, *p* = 0.001	−0.263, *p* = 0.006	−0.216, *p* = 0.023	−0.207, *p* = 0.037
Lawton index	0.151, *p* < 0.001				0.230, *p* = 0.021
DSC vs. STST 10				−0.356, *p* = 0.031	
DSC vs. STST 30				0.494, *p* = 0.002	
DSC vs. TMTA	−0.323, *p* < 0.001	−0.147, *p* = 0.026			−0.224, *p* = 0.034
DSC vs. TMTB	−0.259, *p* < 0.001				
DSC vs. Fall			−0.256, *p* = 0.022		

WC = Waist circumference. WtHR = Waist-to-height ratio. DHR = Daytime heart rate. BMI = Body mass index. STST = Sit-to-stand test. TMT = Trail Making Test. MCS = Mental Health Component Summary. PCS = Physical Component Summary. DSC = Daily steps count.

## Data Availability

The data that support the findings of this study are available from the corresponding author upon reasonable request.

## Abbreviations

The following abbreviations are used in this manuscript:

6MWT	6 Minutes Walking Test
BMI	Body Mass Index
DSC	Daily Step Count
GS	Gait Speed
MCS	Mental Health Component Summary
MVPA	Moderate Vigorous Physical Activity
PA	Physical Activity
PCS	Physical Component Summary
STST	Sit to Stand Test
TMT	Trail Making Test
WC	Waist Circumference
WtRH	Waist to Height Ratio
